# Gut Bacterial Metabolite Urolithin A (UA) Mitigates Ca^2+^ Entry in T Cells by Regulating miR-10a-5p

**DOI:** 10.3389/fimmu.2019.01737

**Published:** 2019-07-31

**Authors:** Shaqiu Zhang, Tamer Al-Maghout, Hang Cao, Lisann Pelzl, Madhuri S. Salker, Marc Veldhoen, Anchun Cheng, Florian Lang, Yogesh Singh

**Affiliations:** ^1^Institute of Preventive Veterinary Medicine, Sichuan Agricultural University, Chengdu, China; ^2^Department of Physiology, University of Tübingen, Tübingen, Germany; ^3^Centre for Clinical Transfusion Medicine, Tübingen University, Tübingen, Germany; ^4^Research Institute of Women's Health, University of Tübingen, Tübingen, Germany; ^5^Instituto de Medicina Molecular, Joâo Lobo Antunes, Faculdade de Medicina da Universidade de Lisboa, Lisbon, Portugal; ^6^Institute of Medical Genetics and Applied Genomics, Tübingen University, Tübingen, Germany

**Keywords:** CD4^+^ T cells, urolithin A, SOCE, miR-10a-5p, Orai1, STIM1/2, bacterial metabolite

## Abstract

The gut microbiota influences several biological functions including immune responses. Inflammatory bowel disease is favorably influenced by consumption of several dietary natural plant products such as pomegranate, walnuts, and berries containing polyphenolic compounds such as ellagitannins and ellagic acid. The gut microbiota metabolizes ellagic acid resulting in the formation of bioactive urolithins A, B, C, and D. Urolithin A (UA) is the most active and effective gut metabolite and acts as a potent anti-inflammatory and anti-oxidant agent. However, whether gut metabolite UA affects the function of immune cells remains incompletely understood. T cell proliferation is stimulated by store operated Ca^2+^ entry (SOCE) resulting from stimulation of Orai1 by STIM1/STIM2. We show here that treatment of murine CD4^+^ T cells with UA (10 μM, 3 days) significantly blunted SOCE in CD4^+^ T cells, an effect paralleled by significant downregulation of Orai1 and STIM1/2 transcript levels and protein abundance. UA treatment further increased miR-10a-5p abundance in CD4^+^ T cells in a dose dependent fashion. Overexpression of miR-10a-5p significantly decreased STIM1/2 and Orai1 mRNA and protein levels as well as SOCE in CD4^+^ T cells. UA further decreased CD4^+^ T cell proliferation. Thus, the gut bacterial metabolite UA increases miR-10a-5p levels thereby downregulating Orai1/STIM1/STIM2 expression, store operated Ca^2+^ entry, and proliferation of murine CD4^+^ T cells.

## Introduction

Polyphenolic compounds are potential anti-inflammatory dietary agents (1, 2). Ellagitannin-rich food products and medicinal plants favorably influence inflammatory bowel disease (3). *In vivo* studies from an animal model of colitis (inflammatory bowel disease) indicate that ellagitannin-containing food products can be especially effective in modulating intestinal inflammation (4). The administration of pomegranate, raspberry, strawberry, and almond preparations was shown to ameliorate the histological derangements of chemically induced inflammation in gut mucosa, an effect accompanied by decreased infiltration of immune cells, blunted expression of pro-inflammatory factors, and the inhibition of inflammation associated molecular pathways (4–7).

The bioavailability of ellagitannins and ellagic acid is, however, rather limited and the substances are metabolized by the gut microbiota yielding bioactive molecules including various urolithins compounds such as urolithin A, B, C, and D (8) that are more readily absorbed than the original polyphenols (9). Urolithin A (UA) is the most abundantly present metabolite in the mouse gut after consumption of pomegranate husks or extract, whereas in humans different ellagitannins sources leads to different urolithins compounds including A, B, and C being formed (9). Urolithins circulate in plasma as glucuronide and sulfate conjugates at concentrations in the range of 0.2–20 μM and it is proposed that conjugation of UA to UA-glucuronide dampens its biological activity *in vivo* (9, 10). However, a recent study suggested that the process of tissue deconjugation especially within the intestinal tract (in a systemic inflammation rat model) allows free availability of UA in inflammatory micro-environmental sites and could thus, have beneficial effects on inflammatory bowel disease or in colon cancer (10, 11). Furthermore, UA metabolites have shown to elicit a potent anti-aging property in *C. elegens* by inducing mitophagy (12). Previous seminal studies have indicated that gut metabolites such as short chain fatty acids (SCFAs) derived from dietary fibers affect the development and function of regulatory T cells and effector T cells (13–16). The characterization of these metabolites produced from polyphenols by gut microbiota is of great clinical interest due to their antioxidant and anti-inflammatory activities (5). Thus, gut metabolites in particular UA, which has an anti-inflammatory property in inflammatory bowel disease and, improves the gut permeability could indeed modify function and activity of immune cells including adaptive immune T cells (4, 17).

CD4^+^ T cell activation relies on an increase of intracellular Ca^2+^ (18, 19). In brief, T-cell receptor (TCR) engagement leads to activation of different signal transduction pathways that cause a rapid release of Ca^2+^ from the endoplasmic reticulum (ER) (20–23). In the quiescent state of T cells, Ca^2+^ is deposited in the ER and sensed by two proteins namely stromal cell-interaction molecule (STIM) 1 and 2 proteins (24). Stimulation of the TCR causes the production of inositol triphosphate (IP_3_) and this signaling molecule binds to IP_3_ receptors at the ER which trigger release of Ca^2+^ into the cytosol (18). The depletion of the ER Ca^2+^ stores leads to store operated Ca^2+^ entry (SOCE) which is accomplished by activation of calcium release-activated calcium (CRAC) channel protein Orai1 by the Ca^2+^ sensing STIM1/2 (18, 25–28). Ca^2+^ influx through Orai1 in T cells depends on a negative membrane potential that provides the electrical driving force for Ca^2+^ entry (18, 27, 29–31). Ca^2+^ entry is required for full triggering of T-cell activation and proliferation, which involves expression of a large number of activation-associated genes (31).

T cell activation is modified by several microRNAs (miRNAs), which are post-transcriptional gene regulators (32–35). A recent study suggested that absence of miRNAs processing enzyme *dicer* inhibits the Ca^2+^ influx in naïve and activated T cells (36). Polyphenols such as green tea induce miR-15b which negatively affects the influx of Ca^2+^ inside the cells by regulating the Ca^2+^ sensing proteins STIM2 and Orai1 (37). However, whether gut metabolites such as urolithins—UA or urolithin B (UB) influence miRNAs thus regulating the physiological functions of CD4^+^ T cells remains unknown.

In this study, we found a completely novel role of urolithins (especially UA) in the regulation of miRNAs expression, SOCE and proliferation of murine CD4^+^ T cells. Our results suggest that in CD4^+^ T cells, UA decreases the expression of Orai1 and STIM1/2 thus compromising SOCE. In addition, we show that UA increases expression of miR-10-5p in a dose-dependent manner, which in turn reduces Orai1 and STIM1/2 transcript and protein levels thus blunting SOCE. Moreover, UA treatment decreased proliferation of CD4^+^ T cells. Thus, the present observations uncover a novel action of UA, i.e., the upregulation of miR-10a-5p with subsequent downregulation of store operated Ca^2+^ influx in CD4^+^ T cells.

## Results

### UA Attenuates Store Operated Ca^2+^ Entry (SOCE)

**Figure 1:**
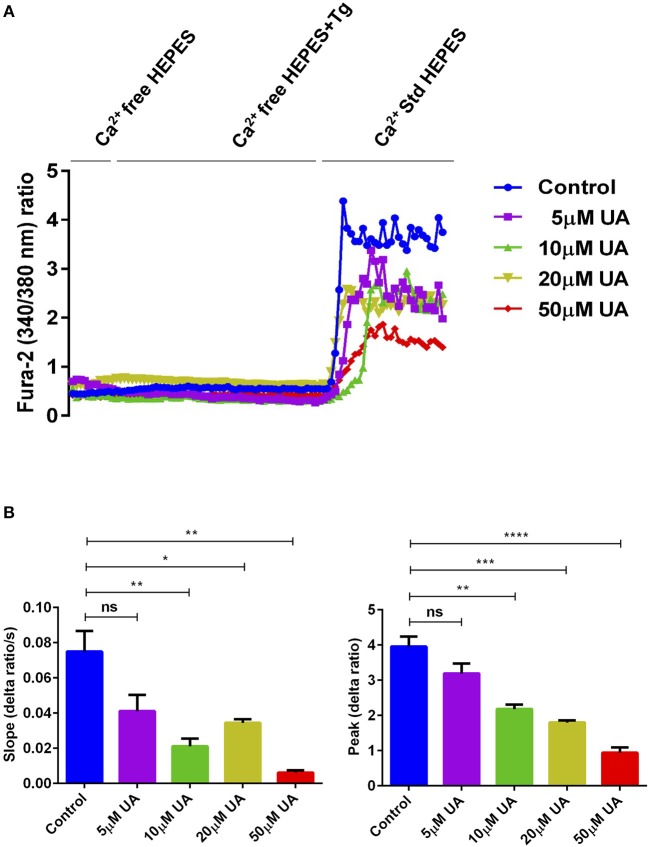
UA treatment significantly decreased SOCE in activated CD4^+^ T cells. **(A)** Representative tracings showing the 340/380 nm fluorescence ratio reflecting cytosolic Ca^2+^ activity in Fura-2, AM loaded and activated (plate bound anti-CD3 and anti-CD28) CD4^+^ T cells which were incubated for 72 h without (control; blue) and with different concentrations of UA (5; purple, 10; green, 20; lemon green or 50; red μM) followed by subsequent exposure to Ca^2+^-free HEPES, additional exposure to sarcoendoplasmatic Ca^2+^ ATPase (SERCA) inhibitor thapsigargin (1 μM; Tg) and re-addition of extracellular Ca^2+^ (Ca^2+^ Std HEPES). **(B)** Arithmetic means ± SEM (*n* = 4) of the slope (left) and peak (right) of the fluorescence ratio change following re-addition of extracellular Ca^2+^ in CD4^+^ T cells incubated for 72 h without (blue bars) and with 5 μM UA (purple bars), 10 μM UA (green bars), 20 μM UA (lemon green bars), and 50 μM UA (red bars). Non significance (ns), ^*^p < 0.05, ^**^p < 0.01, ^***^p < 0.001, ^****^p < 0.0001 indicates statistically significant difference using Student's *t*-test.

Orai1 channels are recruited after being stimulated by STIM1/2 and accomplish SOCE into CD4^+^ T cells which is decisive for T cell activation (18). To quantify the intracellular Ca^2+^ activity ([Ca^2+^]i) and SOCE from control and UA treated CD4^+^ T cells, Fura-2 fluorescence was determined. Activated (plate-bound anti-CD3 and anti-CD28) and unactivated CD4^+^ T cells were used as a control to measure the SOCE in this experimental set up (Supplementary Figure 1A). CD4^+^ T cells were activated for 72 h in the presence of plate-bound anti-CD3 and anti-CD28 (1:2 ratio) and in the presence or absence of UA (5–50 μM) The activated cells were incubated with Fura-2 for 30 min in standard HEPES and washed once with standard HEPES. [Ca^2+^]i was measured first in standard HEPES, which was subsequently replaced by Ca^2+^-free HEPES. Intracellular Ca^2+^ stores were depleted by addition of sarco-/endoplasmic reticulum Ca^2+^ ATPase (SERCA) inhibitor thapsigargin (1 μM) in the nominal absence of extracellular Ca^2+^. The subsequent re-addition of extracellular Ca^2+^ was followed by a sharp increase of [Ca^2+^]i, reflecting SOCE. T cells cultured with UA showed dose-dependent (5–50 μM concentrations) reduction in slope and peak of the [Ca^2+^]i increase (Figure 1). In a similar fashion Urolithin B (UB) tended to decrease in the intracellular Ca^2+^ uptake, however no significant change was observed even at 20 μM concentration (Supplementary Figure 1B). Due to a lack of functional change in the intracellular Ca^2+^ uptake after UB treatment (20 μM), we decided not to pursue the UB study and focussed only on UA in all further experiments.

### UA Downregulates the Expression of Orai1 and STIM1/2

**Figure 2:**
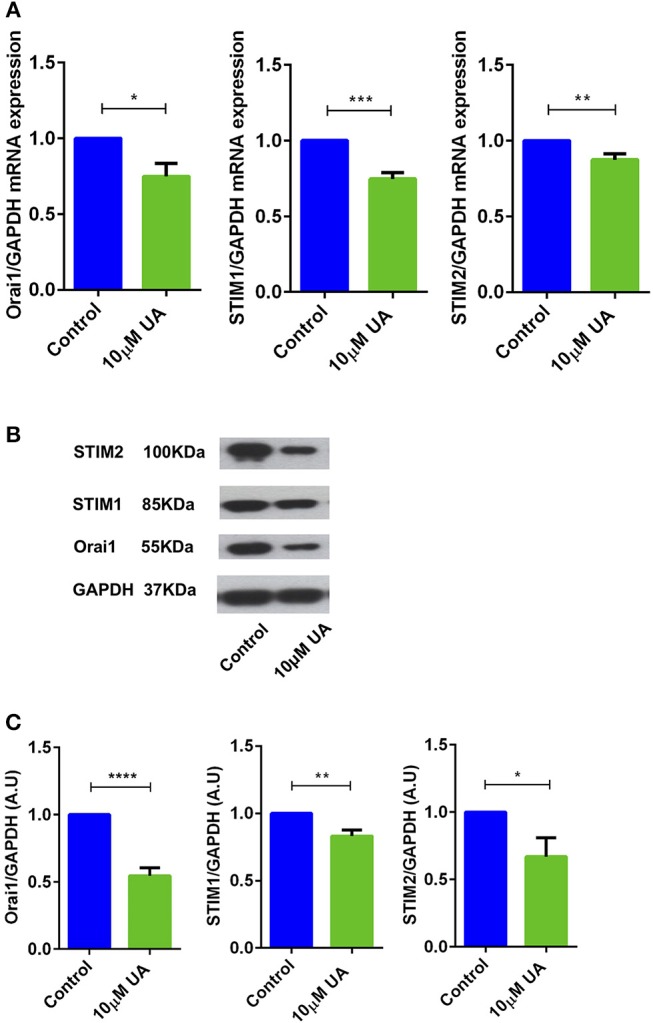
UA significantly decreased Orai1 and STIM1/2 transcripts and protein abundance in CD4^+^ T cells. **(A)** Arithmetic means ± SEM (*n* = 4–7) of Orai1/GAPDH, STIM1/GAPDH and STIM2/GAPDH transcript levels in CD4^+^ T cells following a 72 h incubation without (blue bars) and with (green bars) 10 μM UA.^*^p < 0.05, ^**^p < 0.01, ^***^p < 0.001 indicates statistically significant difference using Student's *t*-test. **(B)** Original Western blots of STIM1/2 as well as of Orai1 with UA treatment. GAPDH was used as a loading control. **(C)** Arithmetic means ± SEM (*n* = 4–7, right panels) of Orai1/GAPDH, STIM1/GAPDH and STIM2/GAPDH protein abundance in CD4^+^ T cells following a 72 h incubation without (control, blue bars) and with (UA, green bars) 10 μM UA. ^*^p < 0.05, ^**^p < 0.01, ^****^p < 0.0001 indicates statistically significant difference using Student's *t*-test.

Utilizing qRT-PCR (Table 1), we further explored whether UA influences Orai1 and/or STIM1/2 transcript levels in CD4^+^ T cells. As illustrated in Figure 2A, treatment of CD4^+^ T cells with 10 μM UA for 72 h significantly decreased Orai1 and STIM1/2 mRNA levels. Western blotting was employed to assess, whether the effect of UA on transcript levels was paralleled by similar effects on protein abundance. UA treatment indeed significantly decreased Orai1 protein and STIM1/2 protein expression (Figures 2B,C).

**Table 1:** Murine qRT-PCR primers.

**Primer name**	**Sequence (5′-3′)**
*Orai1-F*	*5′-CCTGGCGCAAGCTCTACTTA-3′*
*Orai1-R*	*5′-CATCGCTACCATGGCGAAGC-3′*
*STIM1-F*	*5′-ATTGTGTCGCCCTTGTCCAT-3′*
*STIM1-R*	*5′-TGGGTCAAATCCCTCTGAGAT-3′*
*STIM2-F*	*5′-TGTCTGTGTCAAGTTGCCCT-3′*
*STIM2-R*	*5′-TGTCTGGCACTTCCCATTGT-3′*
*GAPDH-F*	*5′-CGTCCCGTAGACAAAATGGT-3′*
*GAPDH-R*	*5′-TTGATGGCAACAATCTCCAC-3′*

### UA Treatment Augments the miR-10a-5p Expression in CD4^+^ T Cells

**Figure 3:**
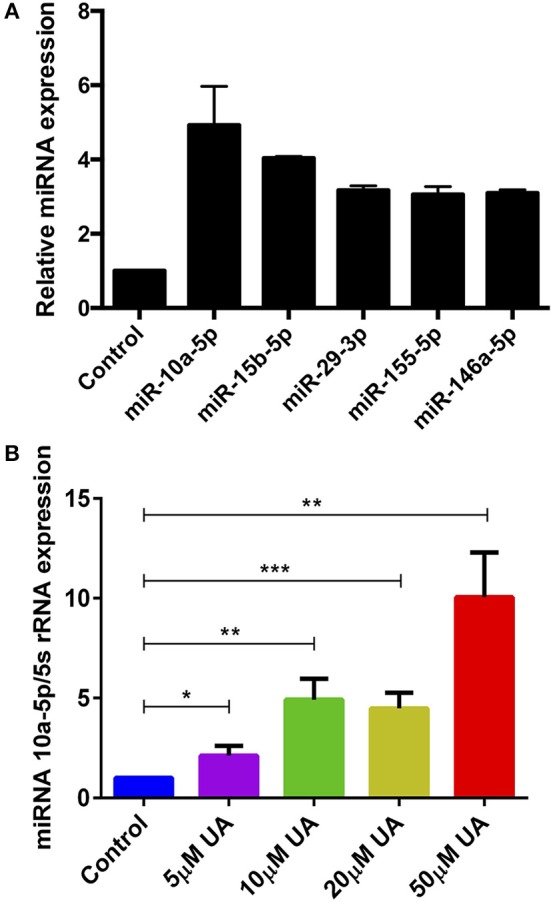
UA treatment significantly increased miR-10a-5p expression in CD4^+^ T cells. **(A)** Arithmetic means ± SEM (*n* = 3) of miR-10a-5p, miR-15b-5p, miR-29a-3p, miR-155-5p, and miR-146a-5p over 5S rRNA transcript levels in CD4^+^ T cells following a 72 h incubation without and with 10 μM UA. **(B)** Arithmetic means ± SEM (*n* = 7) of miR-10a-5p over 5S rRNA transcript levels in CD4^+^ T cells following a 72 h incubation without (blue bars) and with 5 μM UA (purple bars), 10 μM UA (green bars), 20 μM UA (lemon green bars), and 50 μM UA (red bars). ^*^p < 0.05, ^**^p < 0.01, ^***^p < 0.001, indicates statistically significant difference using Student's *t*-test.

Various metabolites and natural plant products are involved in the regulation of miRNAs biogenesis (38). Previously, we have shown that miR-15b was involved in the regulation of STIM2 when treated with green tea polyphenol EGCG (37). Bioinformatics analysis (www.microrna.org, www.targetscan.org, www.mirbase.org) suggested that in addition to miR-15b, several other miRNAs such as miR-10a-5p, miR-29, miR-146 could also modify Ca^2+^ regulating proteins such as Orai1/STIMs. Thus, we next explored whether UA influences the expression of different miRNAs which could be involved in the Ca^2+^ regulation. Therefore, we measured above miRNAs expression in murine CD4^+^ T cells after treatment with UA (10 μM) utilizing the miR-qRT-PCR method. We identified that miR-10a-5p was abundantly present compared with other miRNAs (miR-15b-5p, miR-29a-3p, miR-155-5p, and miR-146a-5p) (Figure 3A). We further tested whether treatment with increasing concentrations of UA (5, 10, 20, 50 μM) could upregulate miR-10a-5p expression in murine CD4^+^ T cells. Indeed, our results suggested that the treatment of CD4^+^ T cells with UA resulted a dose-dependent and significant increase of miR-10a-5p abundance (Figure 3B).

### miR-10a-5p Gain and Loss Inversely Affects Orai1 and STIM1/2 Transcript and Protein Levels

**Figure 4:**
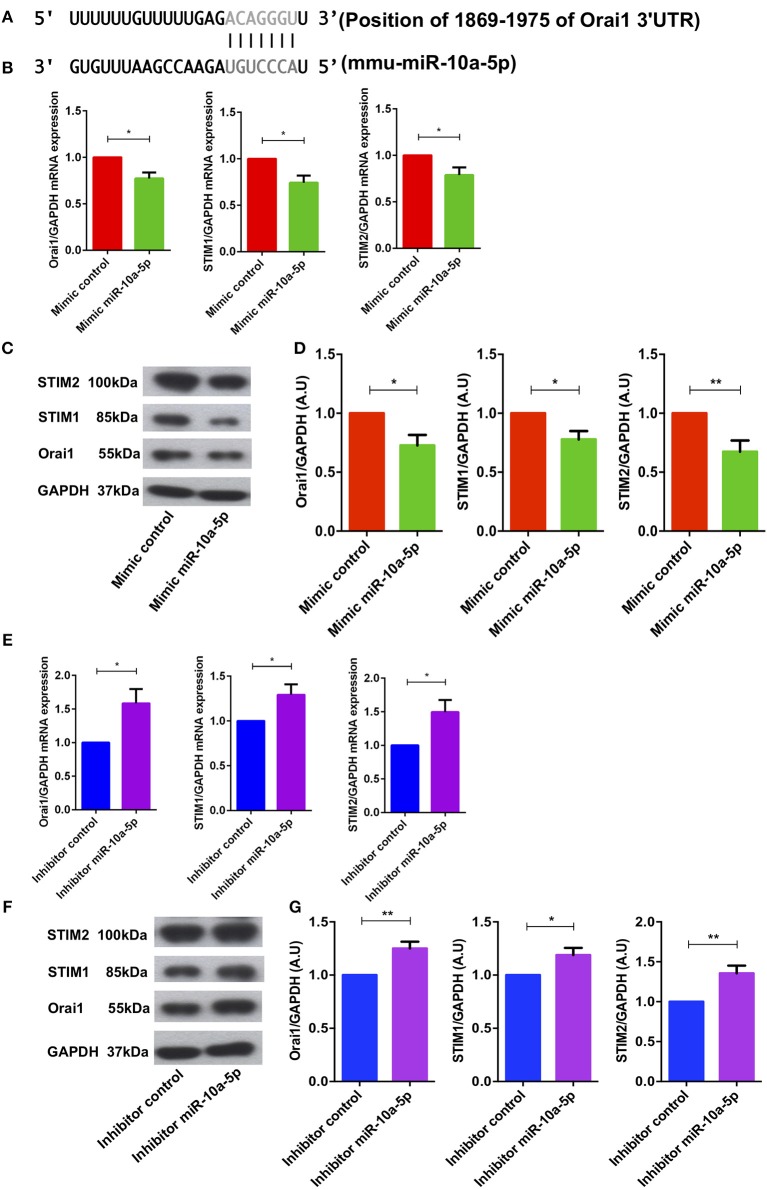
miR-10a-5p mimic over-expression and inhibition significantly decreased and increased Orai1 and STIM1/2 transcript levels and protein abundance in CD4^+^ T cells, respectively. **(A)** Schematic diagram illustrating the putative binding sites of miR-10a-5p matching with the Orai1 3′-untranslated region (3′-UTR) with seed sequence. **(B–D)** Arithmetic means ± SEM (*n* = 4) of **(B)** Orai1/GAPDH, STIM1/GAPDH, and STIM2/GAPDH transcript levels in mimic control (red bars) and miR-10a-5p mimic (green bars) transfected CD4^+^ T cells. **(C)** Original Western blots (left panels) and **(D)** arithmetic means ± SEM (*n* = 5, right panels) of Orai1/GAPDH, STIM1/GAPDH, and STIM2/GAPDH protein abundance in CD4^+^ T cells in mimic control (red bars), miR-10a-5p mimic (green bars) transfected CD4^+^ T cells. ^*^p < 0.05, ^**^p < 0.01 indicates statistically significant difference using Student's *t*-test. **(E–G)** Arithmetic means ± SEM (*n* = 5) of **(E)** Orai1/GAPDH, STIM1/GAPDH and STIM2/GAPDH transcript levels in inhibitor control (blue bars) and miR-10a-5p inhibitor (purple bars) transfected CD4^+^ T cells. **(F)** Original Western blots (left panels) and **(G)** arithmetic means ± SEM (*n* = 5, right panels) of Orai1/GAPDH, STIM1/GAPDH, and STIM2/GAPDH protein abundance in CD4^+^ T cells in inhibitor control (blue bars), miR-10a-5p inhibitor (purple bars) transfected CD4^+^ T cells.^*^p < 0.05, ^**^p < 0.01 indicates statistically significant difference using Student's *t*-test.

Bioinformatics analysis revealed that miR-10a-5p has a strong binding site in the 3′ untranslated region (3′UTR) of Orai1 and is thus a potential regulator of Ca^2+^ entry (Figure 4A). To ascertain whether miR-10a-5p influenced Orai1 and/or STIM1/2 transcription, we transfected CD4^+^ T cells with a negative mimic or a miR-10a-5p mimic and measured Orai1 and STIM1/2 transcript levels. The qRT-PCR data showed a profound and significant downregulation of both Orai1 and STIM1/2 transcript levels following miR-10a-5p transfection (Figure 4B). The decrease of transcript levels was paralleled by similar alterations of protein levels. As apparent from Western blotting figure, miR-10a-5p overexpression was followed by downregulation of Orai1 and STIM1/2 protein abundance (Figures 4C,D). Transfection of miR-10a-5p thus decreased Orai1 and STIM1/2 expression both at transcript and protein levels. Conversely, inhibition of miR-10a-5p was followed by a significant increase of both Orai1 and STIM1/2 transcript levels (Figure 4E) and protein abundance (Figures 4F,G).

### miR-10a-5p Overexpression and Inhibition Inversely Influence SOCE in CD4^+^ T Cells

**Figure 5:**
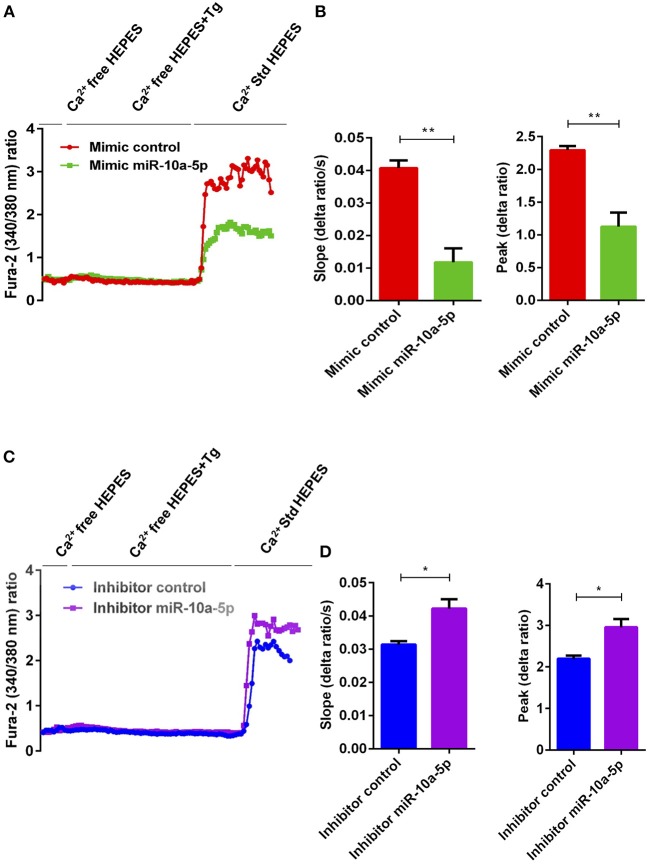
miR-10a-5p overexpression and inhibition lead to loss-of-function and gain-of-function of SOCE in activated CD4^+^ T cells, respectively. **(A)** Representative tracings showing the 340/380 nm fluorescence ratio reflecting cytosolic Ca^2+^ activity in Fura-2/AM loaded negative mimic control (red), and miR-10a-5p mimic (green) transfected CD4^+^ T cells following exposure to Ca^2+^-free HEPES, additional exposure to thapsigargin (1 μM; Tg), and re-addition of extracellular Ca^2+^ (Ca^2+^ Std HEPES). **(B)** Arithmetic means ± SEM (*n* = 3) of the slope (left) and peak (right) of the fluorescence ratio change following re-addition of extracellular Ca^2+^ in negative mimic control (red bars) and miR-10a-5p mimic (green bars) transfected CD4^+^ T cells. ^**^p < 0.01 indicates statistically significant difference using Student's *t*-test. **(C)** Representative tracings showing the 340/380 nm fluorescence ratio reflecting cytosolic Ca^2+^ activity in Fura-2/AM loaded inhibitor control (blue), and miR-10a-5p inhibitor (purple) transfected CD4^+^ T cells following exposure to Ca^2+^-free HEPES, additional exposure to thapsigargin (1 μM) and re-addition of extracellular Ca^2+^ (Ca^2+^ Std HEPES). **(D)** Arithmetic means ± SEM (*n* = 3) of the slope (left) and peak (right) of the fluorescence ratio change following re-addition of extracellular Ca^2+^ in inhibitor control (blue bars), and miR-10a-5p inhibitor (purple bars) transfected CD4^+^ T cells. ^*^p < 0.05 indicates statistically significant difference using Student's *t*-test.

To determine, whether the downregulation of Orai1 and STIM1/2 expression following miR-10a-5p overexpression was paralleled by a similar decrease of SOCE, both control mimic and miR-10a-5p mimic transfected CD4^+^ T cells were activated for 3 days in the presence of plate-bound anti-CD3 and anti-CD28 (1:2 ratio). Ca^2+^ entry was measured at day 3 after transfection of miR-10a-5p overexpression using miRNAs mimic. The activated cells were loaded with Fura-2 for 30 min in standard HEPES and washed once with standard HEPES. [Ca^2+^]_i_ was measured first in standard HEPES, which was subsequently replaced by Ca^2+^-free HEPES. In a next step the intracellular Ca^2+^ stores were depleted by addition of thapsigargin (1 μM) in the nominal absence of extracellular Ca^2+^. The subsequent re-addition of extracellular Ca^2+^ was followed by a sharp increase of [Ca^2+^]_i_. Both, slope and peak of the [Ca^2+^]_i_ increase were significantly lower in miR-10a-5p mimic transfected than in control mimic transfected cells (Figures 5A,B). Thus, our data suggest that overexpression of miR-10a-5p contributes to the downregulation of Orai1 and STIM1/2 expression following UA treatment. Conversely, inhibition of miR-10a-5p augments significantly both slope and peak of the [Ca^2+^]_i_ increase following Ca^2+^ re-addition (Figures 5C,D). Our gain-of-function and loss-of-function data suggested that indeed miR-10a-5p is a powerful regulator of SOCE.

### Effect of UA on Cell Proliferation

**Figure 6:**
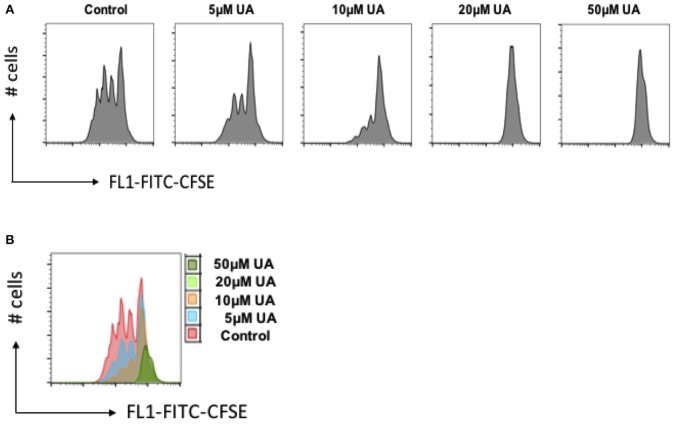
UA decreased cell proliferation of CD4^+^ T cells. **(A)** CD4^+^ T cells were stained with CFSE dye before activation with anti-CD3/anti-CD28 and cultured in the presence of (5–50 μM) UA for 3 days. Cell proliferation was measured by flow cytometry. Data are representative for 3 independent experiments. X-axis represents the CFSE dye whereas y-axis represents cell numbers (# no. of cells). **(B)** Overlays plot of cell proliferation with different concentrations of UA. X-axis represents the CFSE dye whereas y-axis represents cell numbers (# no. of cells).

As stimulation of SOCE is involved in the signaling triggering T-cell proliferation (31), cell proliferation was quantified using the dye CFSE. As illustrated in Figure 6, cell proliferation was significantly decreased in the presence of 10 μM UA.

## Discussion

Ellagitannin-rich food has beneficial effects on inflammatory bowel disease and other inflammatory diseases (3). However, the bioavailability of these compounds (ellagitannins and ellagic acid) is limited and the compounds must be metabolized by the gut microbiota to produce bioactive molecules that can be easily absorbed (9). Ellagic acids, with the help of gut bacteria, are converted into different Urolithins (A-D) (9). The gut commensal bacteria are key component of our body as they are involved in the nutrient uptake and digestion of dietary molecules/fibers which can modify and regulate the function and behavior of immune cells (39, 40). The most common gut bacterial metabolites such as SCFAs (propionic acid, acetic acid, and butyric acid) can positively contribute to generation of the adaptive immune regulatory T cells (13–16). Furthermore, the bacterial metabolite acetate is involved in the reduction of inflammation in Type 1 diabetes model by reducing the autoimmune CD8^+^ T cells (15). The role of urolithins (UA or UB) in T cell activation remained elusive. In this report, we reveal that UA can suppress SOCE by modulating Orai1/STIMs regulating miR-10a-5p, thus affecting Ca^2+^ sensitive cellular functions including CD4^+^ T cell proliferation and activation. Earlier *in vivo* studies suggested UA can suppress the inflammation in mouse and rat models of colitis (4, 17), however, no T cells mediated mechanisms have been described. Hence, UA could potentially envisage as an inflammation reduction agent for T cells, further *in vivo* studies are warranted to confirm this in other inflammatory autoimmune diseases such as Type 1 diabetes, Multiple sclerosis, and Rheumatoid arthritis, etc.

High levels of intracellular Ca^2+^ are necessary to maintain numerous functions of T cells such as the interaction between a T-cell and antigen-presenting cell (APC) that leads to formation of the specialized contact surface known as the immunological synapse and activation of different transcription factors (18, 24, 28, 41, 42). Several hours of oscillating Ca^2+^ influx are required to complete the T-cell activation program, which involves expression of a large number of activation-associated genes (31). Our observations describe that bacterial metabolite product UA is a negative regulator of SOCE into murine CD4^+^ T cells, an effect paralleled by downregulation of Orai1/STIM1/2 expression. Thus, the present observations also uncover a completely novel mechanism accounting for the effect of UA on SOCE, i.e., the upregulation of miR-10a-5p, which in turn downregulates Orai1 and STIM1/2 transcript and protein levels as well as SOCE. Thus, UA changes the post-transcriptional machinery of the key players accomplishing SOCE in CD4^+^ T cells, i.e., Orai1 and STIM1/2.

Recently published studies in patients with inflammatory bowel disease suggested that butyrate-producing gut bacterium *Faecalibacterium prausnitzii* is found to be reduced in active IBD patients over a span of more than 2 years (in a follow up study) and differences in the microbiome over time in individual patients were greatest in the presence of ongoing intestinal inflammation (43–45). In animal models, enteric pathogens such as *Salmonella typhimurium* and *Citrobacter rodentium* promote intestinal inflammation by modifying the TGF-β/Smad signaling pathways (46). The bacterial dysbiosis is appeared to be the prime driver of inflammatory process in various diseases. However, the host system has evolved to defend itself from ongoing inflammation induced by gut bacterial dysbiosis by production of bactericidal lectin RegIIIβ, which can kill certain Gram-positive and Gram-negative bacteria, gut commensal microbiota and enteropathogenic bacteria, however, certain bacterial species such as *Salmonella Typhimurium* is resistant to RegIIIβ bactericidal activity (47). Therefore, external intervention or therapy is required to resolve the ongoing inflammation. Many xenobiotic drugs can modulate the inflammation however, these drugs/chemical need toxicological risk assessment for human health and ecology before it can be release for public use as our commensal gut microbiota can convert them into toxic metabolites (48). Supplementation with specific commensal microbiota strains or their specific metabolites could be an alternative for inflammation therapy as they do not require to be rejected by the host. UA could have a great potential as it is a bacterial metabolite and safe to use for inflammation resolution (49). Emerging evidence indicates that UA is involved in the regulation of inflammatory pathways, cell cycle and cell death (1, 12, 50). In keeping with this conjecture, in inflammatory bowel disease rat-models UA reduced inflammation (4). However, some studies suggested that ingested UA can be modified into glucuronide and sulfate conjugates *in vivo*, therefore, bioavailability of UA can be compromised (9, 10). Interestingly, new evidence has pointed to the process of tissue deconjugation in inflammatory microenvironmental sites, which convert the UA-glucuronide conjugate into the free-form of UA (10, 11). Moreover, it has also recently been shown that UA and UAS03 (a potent synthetic analog of UA) are involved in the improving the gut barrier functions by activation of aryl hydrocarbon receptor dependent pathways to upregulate epithelial tight junction proteins as well-induced an anti-inflammatory environment to prevent the chemically-induced colitis in a pre-clinical mouse model (17). These studies further substantiate the claim that bacterial metabolite UA could possibly be an ideal candidate to alleviate inflammation in the gastrointestinal tract. Some other groups have reported that UA can counteract the growth of cancer cells and could thus be used as anti-cancer agent (1, 50). It is tempting to speculate that UA interferes similarly with tumor cell proliferation and inflammation by downregulating SOCE. However, further validations are needed to define precise mechanisms involved in effects of UA on tumor cells and inflammation.

Recently, we have demonstrated that miRNAs processing protein *dicer* is involved in the regulation of SOCE in CD4^+^ T cells (36). Thus, identifying the role of individual miRNAs which could be involved in the regulation of Ca^2+^ pathways, opens up a new avenue for therapeutic intervention. Using bioinformatics tools, we found that miR-10-5p could regulate Orai1 proteins and modify STIM1/2 expression. Previous studies have reported that miR-10a-5p is involved in the development and function of regulatory T cells (51). Our results reveal that miR-10a-5p is upregulated after treatment with UA in T cells. Thus, miR-10a-5p appears to be involved in the regulation of Ca^2+^ entry and thus Ca^2+^ sensitive cellular functions such as gene expression, proliferation, cell motility, and cytokine expression. However, a role of other miRNAs or further signaling pathways cannot be excluded and further *in vivo* evidence is warranted to elucidate the physiological connection between UA and suppression of CD4^+^ T cell proliferation via miR-10a-5p-induced reduction of Ca^2+^ influx.

In conclusion, the present observations reveal a completely novel role of gut bacterial metabolite UA in the regulation of Ca^2+^ entry into CD4^+^ T cells leading to suppression of activation of CD4^+^ T cells. UA upregulates the expression of miR-10a-5p which in turn decreases SOCE by downregulating Orai1 and STIM1/2 expression. Thus, our results suggest that upregulation of miR-10a-5p by UA restrains SOCE in murine CD4^+^ T cells and UA could be used as a natural immune-suppressant during various inflammatory disorders including inflammatory bowel disease.

## Materials and Methods

### Mice

Naïve CD4^+^ T cells were isolated from C57BL/6 mice (male and female) between 8 and 16 weeks of age. All the animals were kept in standard housing conditions with 12± dark/light cycle and fed on a standard chow diet and had *ad lib* access to drinking water.

### Naïve CD4^+^ T Cell Isolation and Culture

Naïve CD4^+^ T cells were purified from spleen and lymph nodes of C57BL/6 mice using the MagniSort® Mouse naïve T cell Enrichment kit (#8804-6824-74, eBioscience, USA) as described by the manufacturer. Purified naïve CD4^+^ T cells were cultured in plate-bound anti-CD3 (#16-0031-85, eBioscience, USA)/anti-CD28 (#16-0281-85, eBioscience, USA) Abs at a 1:2 ratio (1 μg/ml anti-CD3 and 2 μg/ml anti-CD28) in the presence or absence of 5–50 μM UA (#1143-70-0, Santa Cruz Biotechnology, USA, purity ≥ 94% HPLC) and 5–50 μM Urolithin B (UB; #SML1649-10MG, Sigma, Germany) for 72 h. Both the compounds UA and UB were dissolved in DMSO (#D8418, Sigma, Germany) to make the 10 mM concentrations and appropriate amounts were used for the experiments as described in the respective figures.

### Intracellular Calcium Measurement

Intracellular Ca^2+^ activity was measured using Fura-2-AM (#F1221, Molecular Probes, USA). Fluorescence measurements were performed using an inverted light incidence fluorescence phase-contrast microscope (Axiovert 100, Zeiss, Germany). Cells were excited alternatively at λ = 340 or 380 nm and the light deflected by a dichroic mirror into either the objective (Fluar 40 × /1.30 oil, Zeiss, Germany) or a camera (Proxitronic, Germany). Emitted fluorescence intensity recorded at λ = 505 nm and data were acquired by using specialized computer software (Metafluor, Universal Imaging, USA) (52).

Activated T cells (3 days) were loaded with 2 μM Fura-2-AM for 30 min at 37°C in a CO_2_ incubator. To measure SOCE, changes in cytosolic Ca^2+^ activity ([Ca^2+^]i) were monitored following depletion of the intracellular Ca^2+^ stores. In short, [Ca^2+^]_i_ was measured using Ca^2+^ containing standard HEPES buffer [125 mM/L NaCl, 5 mM/L KCl, 1.2 mM/L ${\text{MgSO}}_{4}^{*}$7H_2_O, 32.2 mM/L HEPES, 2 mM/L Na_2_${\text{HPO}}_{4}^{*}$2H_2_O, 5 mM/L Glucose, 1 mM/L ${\text{CaCl}}_{2}^{*}$2H_2_O; pH = 7.4] for 2 min and then changed to Ca^2+^-free HEPES buffer [125 mM/L NaCl, 5 mM/L KCl, 1.2 mM/L ${\text{MgSO}}_{4}^{*}$7H_2_O, 32.2 mM/L HEPES, 2 mM/L Na_2_${\text{HPO}}_{4}^{*}$2H_2_O, 5 mM/L Glucose, 0.5 mM/L EGTA; pH = 7.4] for 3 min. In the absence of Ca^2+^, the intracellular Ca^2+^ stores were depleted by inhibition of the sarcoendoplasmatic Ca^2+^ ATPase (SERCA) by 1 μM thapsigargin (#67526-95-8, Sigma, Germany) and [Ca^2+^]_i_ was measured for another 5 min. In the following, Ca^2+^ containing HEPES buffer was added for 5 min, which allowed assessing the SOCE.

### Transfection of CD4^+^ T Cells by miR-10a-5p

Naïve CD4^+^ T cells were seeded on a coated 24-well plate. Naïve T cells were transfected with miR-negative mimic (#479903), miR-10a-5p mimic (#471928), miR-10a-5p inhibitor (#4100036) (Exiqon, Denmark) using DharmaFECT3 (#T-2003-01, Dharmacon, USA) as recommended by manufacture's guidelines. Briefly, naïve T cells were prepared in antibiotic free cell buffer and 0.75 × 10^6^-1 × 10^6^ cells per well-cultured in the presence of 500 μl of R-10 medium (RPMI 1640 #61870-010, Life technologies, USA) medium supplemented with 10% fetal bovine serum (#10270-106, life technologies, USA), 1% L-Glutamine (#G7513 200 mM solution, Sigma, Germany), 1% penicillin/streptomycin (#P4333, Sigma, Germany), and 0.1% 2-Mercaptoethanol (#31350-010, Life technologies, USA). Whilst plating the cells, 2 μl of 50 μM stock concentration of non-targeting miRNA-negative mimic, miR-10a-5p-mimic or miR-10a-5p-inhibitor were added to 8 μl of antibiotic free RPMI1640 medium and the cells incubated for 5 min in tube 1, respectively. In tube 2, 0.5 μl of DharmaFECT3 was added to 9.5 μl of antibiotic free RPMI1640 medium. The content of tube 1 was added to tube 2 and incubated for additional 20 min. After 20 min of incubation, the reaction mixture from tube 2 was added to corresponding wells to control and miR-10a-5p-mimic or miR-10a-5p-inhibitor wells. Cells were further incubated for additional 72 h and used for qRT-PCR, immunoblotting, and determination of SOCE.

### mRNA and miRNA qRT-PCR

Total RNA including miRNAs was extracted from CD4^+^ T cells using miRNAeasy Kit (#217004, Qiagen, Germany). The mRNA (1 μg) and miRNAs (100 ng) were separately reverse transcribed using Superscript III First-Strand synthesis system (#18080-51, Invitrogen, Germany) and miRNA universal cDNA synthesis kit II (#203301, Exiqon, Denmark) for reverse transcript PCR (RT-PCR) and subsequent real-time quantitative PCR (qRT-PCR). Detection of gene expression was performed with KapaFast-SYBR Green (#KAPBKK4606, Peqlab, Germany) and measurements were performed on a BioRad iCycler iQ^TM^ Real-Time PCR Detection System (Bio-Rad Laboratories). The relative expression levels of mRNAs were normalized to that of *GAPDH*, whereas the relative expression levels of miRNAs were normalized to that of 5S rRNA. The following murine primers were used to detect *Orai1, STIM1*, and S*TIM2* expression (53).

For amplification of different miRNAs, hsa-miR-10a-5p LNA™ PCR primer set (#204778, Exiqon, Denmark), hsa-miR-15b-5p LNA™ PCR primer set (#204243), hsa-miR-29a-3p LNA™ PCR primer set (#204698), hsa-miR-146a-5p LNA™ PCR primer set (#204688), mmu-miR-155-5p LNA™ PCR primer set (#205930), and reference 5S rRNA primer set (#203906) were used and the reaction was set up as recommended by Exiqon or described earlier (54–56).

### Immunoblotting

CD4^+^ T cells were activated in presence of anti-CD3 (1μg/ml)/anti-CD28 (2μg/ml) and treated with 10 μM UA. After 72 h of activation and treatment, CD4^+^ T cells were washed once with PBS, counted and equal amounts cells were taken for cell lysis using H_2_O and 2 × Lammelli's Buffer. Proteins were denatured at 95°C for 5–10 min and stored at −20°C. Sample proteins were loaded on 8 or 10% gel depending on protein size and run at 80 V until crossing of stacking gel then voltage was increased to 120 V during the separation phase and total gel run for 90–100 min. Proteins were electro-transferred onto PVDF membranes. Membranes were probed with the indicated primary antibodies for Orai1 (1:1000; #13130-1-AP, Proteintech, United Kingdom), STIM1 (1:1000; #5668S, Cell Signaling Technology), STIM2 (1:1000; #4917S, Cell Signaling Technology), and GAPDH (1:2000; #5174S, Cell Signaling Technology), followed by HRP-conjugated secondary antibodies (1:1000; #7074P2, Cell Signaling Technology, Germany). Membranes were washed and visualized with enhanced chemiluminescent HRP substrate (#R-03031-D25 and R-03025-D25, advansta, USA). Data were analyzed by ImageJ software (https://imagej.nih.gov/ij/).

### CFSE Staining

The proliferation of CD4^+^ T cells was detected by CellTrace™ CFSE Cell Proliferation Kit (#C34554, eBioscience, USA). Briefly, cells were washed with PBS (#D8537, Sigma, Germany) once, stained with CellTrace™ CFSE (1:1000 dilution) and re-suspended gently, incubated at 37°C for 15 min in the dark, then washed with R-10 medium twice and activated as described in *CD4*^+^ *T cell isolation and culture* in Materials and Methods above. After 72 h, cells were collected to perform the flow cytometry. Data were analyzed by Flowjo software (FLOWJO LLC-BD, USA).

### Ca^2+^ Measurement by Flow Cytometry

The CD4^+^ T cells were activated and treated as described earlier with UA (10 μM) and UB (20 μM) for 3 days. After 3 days, cells were stained with Fluo-4 dye (#F14201; Thermofisher, Germany) as described by manufacture's protocol and described earlier (36). Fluo-4 is an analog of Fluo-3 with the two chlorine substituents replaced by fluorines, which results in increased fluorescence excitation at 488 nm and consequently higher fluorescence signal levels. The stained cells were acquired on FACS Calibur™ and data were analyzed by Flowjo software.

### Statistics

Data are provided as means ± SEM, *n* represents the number of independent experiments. All data were tested for significance using unpaired Student's *t*-test. Data were analyzed by Excel 2010 or GraphPad Prism Software, USA. *P* value ≤0.05 was considered statistically significant.

## Data Availability

The raw data supporting the conclusions of this manuscript will be made available by the authors, without undue reservation, to any qualified researcher.

## Ethics Statement

All experiments were performed according to the EU Animals Scientific Procedures Act and the German law for the welfare of animals. All procedures were approved by local government authorities (Regierungspräsidium Tübingen according to §4 animal welfare act on 29/05/2015 and 20/02/2017) of the state of Baden-Württemberg, Germany.

## Author Contributions

SZ, TA-M, HC, LP, MS, and YS performed the research and analyzed the data. MV provided the intellectual input, reagents, and tools. AC, FL, and YS designed the study, supervised the project, and wrote the manuscript. All authors edited and approved the final manuscript.

### Conflict of Interest Statement

The authors declare that the research was conducted in the absence of any commercial or financial relationships that could be construed as a potential conflict of interest.
