# Supplementary material for: Gut Bacterial Metabolite Urolithin A (UA) Mitigates Ca2+ Entry in T Cells by Regulating miR-10a-5p
Source: Front Immunol. 2019 Jul 31;10:1737. doi: 10.3389/fimmu.2019.01737 (PMC6685097; doi:10.3389/fimmu.2019.01737)
Supplement: Supplementary file 1 [file Image_1.pdf]

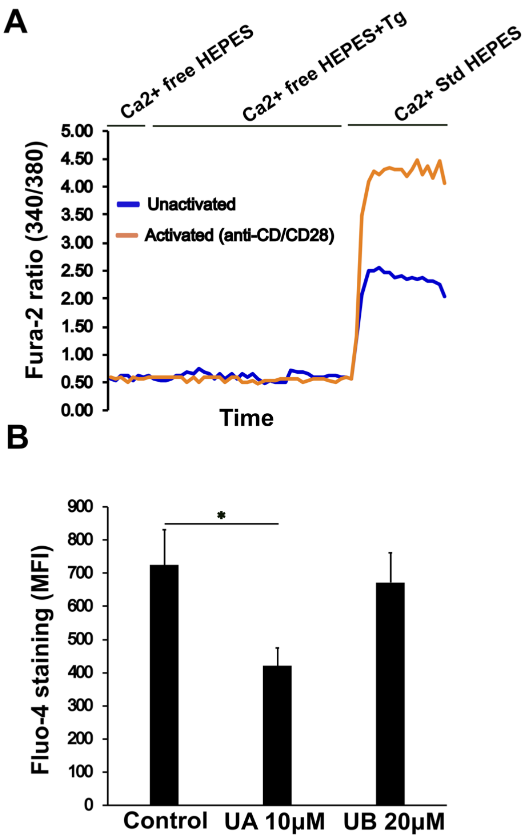

SUPPL. Fig.1: **Urolithins (A and B) treatment decrease Ca^{2+} levels in $CD4^{+}$ T cells**

A. To establish intracellular Ca^{2+} measurements by fluorescence imaging, we used inactivated and 3 days activated $CD4^{+}$ T cells and measured SOCE as described in materials and method section. As expected, unactivated T cells (blue) have less SOCE over activated T cells (red).

B. To test intracellular Ca^{2+} levels after treatment with UA or UB, we used a flow cytometry approach and found that UA (10μM) has significant reduction in Ca^{2+} levels compared with the control (untreated). However, in the case of UB (20μM) treatment, no statistical significance in reduction of Ca^{2+} levels were achieved.

*($p < 0.05$) indicates statistically significant difference using Student’s t-test.
